# Associations of combined genetic and lifestyle risks with hypertension and home hypertension

**DOI:** 10.1038/s41440-024-01705-8

**Published:** 2024-06-24

**Authors:** Masato Takase, Takumi Hirata, Naoki Nakaya, Tomohiro Nakamura, Mana Kogure, Rieko Hatanaka, Kumi Nakaya, Ippei Chiba, Ikumi Kanno, Kotaro Nochioka, Naho Tsuchiya, Akira Narita, Hirohito Metoki, Michihiro Satoh, Taku Obara, Mami Ishikuro, Hisashi Ohseto, Akira Uruno, Tomoko Kobayashi, Eiichi N. Kodama, Yohei Hamanaka, Masatsugu Orui, Soichi Ogishima, Satoshi Nagaie, Nobuo Fuse, Junichi Sugawara, Shinichi Kuriyama, Gen Tamiya, Atsushi Hozawa, Masayuki Yamamoto, the ToMMo investigators

**Affiliations:** 1 Graduate School of Medicine, Tohoku University, Aoba-ku, Sendai, Miyagi Japan; 2 Tohoku Medical Megabank Organization, Tohoku University, Aoba-ku, Sendai, Miyagi Japan; 3 Human Care Research Team, Tokyo metropolitan Institute for Geriatrics and Gerontology, Tokyo, Japan; 4 Kyoto Women’s University, Kyoto, Japan; 5 Tohoku University Hospital, Tohoku University, Aoba-ku, Sendai, Miyagi Japan; 6 Tohoku Medical and Pharmaceutical University, Miyagino-ku, Sendai, Japan; 7 International Research Institute of Disaster Science, Tohoku University, Aoba-ku, Sendai, Miyagi Japan; 8 Suzuki Memorial Hospital, Satonomori, Iwanumashi, Miyagi, Japan; 9 RIKEN Center for Advanced Intelligence Project, Tokyo, Japan

**Keywords:** Hypertension, Lifestyle risk reduction, Polygenic risk score

## Abstract

No study, to our knowledge, has constructed a polygenic risk score based on clinical blood pressure and investigated the association of genetic and lifestyle risks with home hypertension. We examined the associations of combined genetic and lifestyle risks with hypertension and home hypertension. In a cross-sectional study of 7027 Japanese individuals aged ≥20 years, we developed a lifestyle score based on body mass index, alcohol consumption, physical activity, and sodium-to-potassium ratio, categorized into ideal, intermediate, and poor lifestyles. A polygenic risk score was constructed with the target data (*n* = 1405) using publicly available genome-wide association study summary statistics from BioBank Japan. Using the test data (*n* = 5622), we evaluated polygenic risk score performance and examined the associations of combined genetic and lifestyle risks with hypertension and home hypertension. Hypertension and home hypertension were defined as blood pressure measured at a community-support center ≥140/90 mmHg or at home ≥135/85 mmHg, respectively, or self-reported treatment for hypertension. In the test data, 2294 and 2322 participants had hypertension and home hypertension, respectively. Both polygenic risk and lifestyle scores were independently associated with hypertension and home hypertension. Compared with those of participants with low genetic risk and an ideal lifestyle, the odds ratios for hypertension and home hypertension in the low genetic risk and poor lifestyle group were 1.94 (95% confidence interval, 1.34–2.80) and 2.15 (1.60–2.90), respectively. In summary, lifestyle is important to prevent hypertension; nevertheless, participants with high genetic risk should carefully monitor their blood pressure despite a healthy lifestyle.

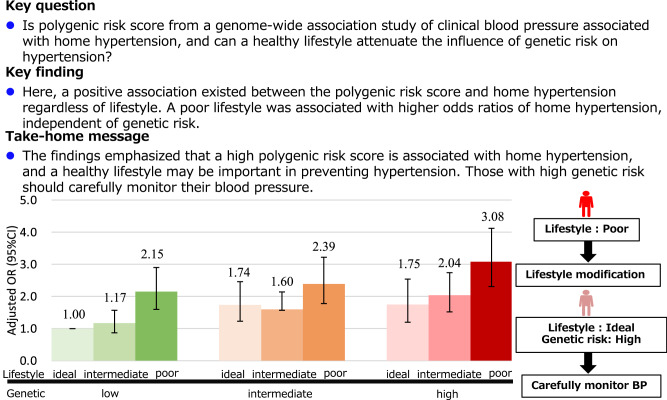

## Introduction

Hypertension is a well-known risk factor for cardiovascular disease (CVD) mortality and morbidity [1]. According to the World Health Organization, an estimated 1.28 billion adults are affected by hypertension worldwide, and 46% of adults with hypertension are unaware of their condition [2]. Therefore, identifying individuals at high risk of developing hypertension and preventing or delaying its onset is important.

Hypertension development is related to genetic and environmental factors [3]. To date, risk factors for hypertension, including alcohol consumption, physical inactivity, obesity, and sodium and potassium intake, are emphasized in many guidelines [4–7]. Recently, large-scale analyses of genome-wide association studies (GWASs) have been conducted, and these studies have identified more than 900 independent hypertension-related genomic regions [8–13]. Subsequently, a polygenic risk score (PRS), which can predict the onset of hypertension, was constructed using single nucleotide polymorphisms (SNPs) associated with GWASs [14, 15].

A few studies have examined the joint associations of genetic susceptibility and lifestyle adherence with hypertension in general populations [16–19]. For example, in the United Kingdom (UK) Biobank Study, high genetic risk was associated with elevated blood pressure (BP) and incident hypertension, independent of lifestyle, whereas poor lifestyle was associated with elevated BP and incident hypertension, regardless of genetic risk [16, 17]. The Henan Rural Cohort study on a Chinese population also showed a combined effect between genetics and lifestyle on BP and hypertension [18]. In the Japan Multi-Institutional Collaborative Cohort (J-MICC) study, the PRS was associated with a higher prevalence of hypertension, independent of lifestyle factors, and lifestyle factors, particularly obesity, were positively associated with the incidence of hypertension, regardless of the PRS [19]. Home BP is recommended in international guidelines for managing and diagnosing hypertension [4–7] as it predicts CVD better than office BP [20–23]. However, whether the PRS, which was constructed based on GWASs for clinical BP, is associated with home hypertension is unknown. Furthermore, it is unknown whether a healthy lifestyle can reduce the influence of genetic risk on hypertension.

Therefore, this study aimed to examine the cross-sectional associations of a combined PRS and healthy lifestyle with the prevalence of hypertension and home hypertension in a large prospective cohort of the Tohoku Medical Megabank Community-based Cohort (TMMCommCohort) Study.

Point of view

- **Clinical relevance:** Polygenic risk score constructed by GWAS of clinical blood pressure is associated with home hypertension as well as hypertension. Conversely, a healthy lifestyle is inversely associated with hypertension regardless of genetic risk.
- **Future direction:** A prospective cohort study is required to clarify whether PRS can predict new-onset hypertension and home hypertension, and to examine the clinical utility of PRS.
- **Consideration for the Asian population:** Adhering to a healthy lifestyle is essential to managing blood pressure regardless of individual’s genetic risk. However, even with a healthy lifestyle, participants at high genetic risk may need to carefully monitor their blood pressure.

## Methods

### TMMCommCohort Study participants

This was a cross-sectional study of individuals aged ≥20 years living in the Miyagi Prefecture, northeastern Japan, included in the TMMCommCohort study. The details have been previously described [24, 25]. Briefly, the TMMCommCohort study was started in May 2013; by March 2016, more than 50,000 participants were recruited through the following two approaches: the type 1 survey (*n* = 41,097 participants), which was performed at specific municipal health check-up sites, and the type 2 survey (*n* = 13,855), which was conducted at assessment centers. Participants provided information on lifestyle and other potentially health-related aspects through blood and urine samples and a mailed self-reported questionnaire. All participants (*n* = 54,952) provided written informed consent for this study. The Institutional Review Board of the Tohoku Medical Megabank Organization approved this study (approval number: 2022-4-047; approval date: June 30, 2022).

**Fig. 1:**
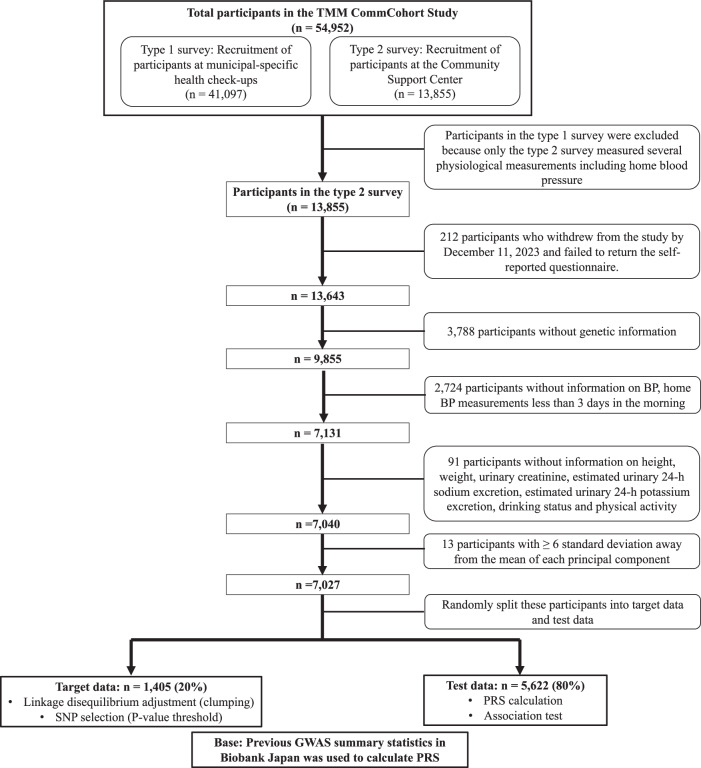
Flowchart of study participants

As several physiological measurements, including home BP, were conducted only in the type 2 survey, we selected participants from this survey group (*n* = 13,855). We excluded participants: (1) who withdrew from the study before December 11, 2023; (2) who failed to return a self-reported questionnaire (*n* = 212); (3) without genetic information genotyped on an Affymetrix Axiom Japonica Array (v2; Affymetrix, Santa Clara, CA, USA) (*n* = 3788); 4) with missing data related to various factors, including BP, home BP measurements for a minimum of 3 days in the morning (*n* = 2724), height, weight, urinary creatinine, estimated urinary 24-h sodium excretion, estimated urinary 24-h potassium excretion, alcohol consumption status, or physical activity (*n* = 91); and 5) with a standard deviation (SD) ≥ 6 for each genetic principal component (*n* = 13). Consequently, 7027 participants fulfilled all inclusion criteria, and their data were analyzed in this study. The data of these participants were randomly categorized into target (*n* = 1405; 20%) and test (*n* = 5622; 80%) data. The target data were used to determine the *P*-thresholds of the best-fit PRS for each trait. The test data were used to examine the associations of the combined PRS and lifestyle score with hypertension or home hypertension (Fig. 1).

### Healthy lifestyle factors

A healthy lifestyle score was constructed based on the following four well-established hypertension risk factors: alcohol consumption status, body mass index (BMI), physical activity, and sodium-to-potassium (Na/K) ratio [4–7]. The Methods, Supplemental Digital Content 1, includes the definitions of alcohol consumption, BMI, physical activity, and Na/K ratio. Subsequently, the overall lifestyle was categorized into ideal (having at least three ideal lifestyle factors), poor (having at least three poor lifestyle factors), or intermediate (having two ideal lifestyle factors).

### BP measurement and ascertainment of hypertension and home hypertension

A trained nurse measured the BP twice in the upper right arm using a digital automatic BP monitor (HEM-9000AI; Omron Healthcare Co., Ltd., Kyoto, Japan) at the community support center after the participants rested for at least 2 min in a sitting position. The mean values of the two recorded measurements were used for analysis. Home BP was measured using a cuff-oscillometric device (HEM-7080IC; Omron Healthcare Co., Ltd.). Participants measured their home BP in a sitting position after resting for at least 5 min in the morning within 1 h of waking; maintaining the arm at heart level during resting; and if applicable, before taking medications for hypertension, eating breakfast, and after urination. The average home BP in the morning for ≥3 days was used for all analyses. Hypertension was defined as systolic BP (SBP)/diastolic BP (DBP) of 140/90 mmHg or higher measured at a community-support center and/or self-reported hypertension treatment. Home hypertension was defined as home SBP/DBP of 135/85 mmHg or higher, or self-reported hypertension treatment [4].

### PRS derived from BioBank Japan (BBJ)

Detailed information about genotyping and quality control in this study is described in the Methods, Supplemental Digital Content 1. We calculated the PRS based on the summary statistics of a previous GWAS for SBP in the BBJ, which is publicly available at the National Bioscience Database Center [26]. Participants included in our study were distinct from those in the BBJ. All SNPs on the X and Y chromosomes were removed from the data to eliminate the possibility of non-autosomal sex effects. PLINK 1.9 (COVID-19 Genomics UK, Cambridge, UK) was used to calculate the PRS using the clumping and thresholding method. Based on a previous study [19], we performed clumping to capture the right level of the causal signal using the following options: --clump-p1 1 --clump-r^2^ 0.1 –clump-kb 250.

After clumping, we calculated the PRS for each individual in the target dataset (*n* = 1405) using various variant sets according to different *P*-value thresholds. The PRS was calculated using the default formula for PRS calculation in PLINK (COVID-19 Genomics UK) (https://choishingwan.github.io/PRS-Tutorial/plink/). As a default setting, we calculated the PRS using the following nine different *P*-value thresholds: 5.0 × 10^−8^, 0.001, 0.01, 0,05, 0.1, 0.2, 0.3, 0.4, and 0.5. Among various PRSs with different numbers of SNPs, we chose the list of variants that showed the best fit (determined using a variance explained with the PRS). The settings of the best-fit PRS for both SBP and home SBP in the target data were a PRS with *P* < 0.001 (Table, Supplemental Digital Content 2). Therefore, we used a PRS constructed using *P* < 0.001 in the test data (*n* = 5622) for the analysis. An overview of the PRS calculations and association study is illustrated in Figure, Supplemental Digital Content 3.

### Statistical analysis

Data were presented as means (SD) or median (interquartile range) for continuous variables and number (percentage) for categorical variables. Participants were classified based on their PRS tertile to analyze the potential association between the PRS and hypertension. Multivariate logistic regression analyses were performed to examine the association of the PRS and lifestyle with the prevalence of hypertension and home hypertension, respectively. Adjusted odds ratios (ORs) and 95% confidence intervals (CIs) were estimated.

Participants were classified into nine groups based on their PRS and lifestyle to examine the combined effect of genetic and lifestyle risks. For hypertension, logistic regression analyses were adjusted for age at inclusion, sex, and the first six principal components (to adjust for population structure). For home hypertension, logistic regression analyses were adjusted for the above model, as well as added measurement seasons of home BP, as home BP was affected by seasonal temperature changes [27, 28].

To examine the influence of genetic risk on the classification performance, we calculated the area under the receiver operating characteristic curve (AUROC) and 95% CI before and after including the PRS in the statistical model, including healthy lifestyle score, using logistic regression analysis. The AUROCs were compared using the DeLong test.

Additionally, to rule out the influence of hypertension treatment, we excluded participants who were receiving treatment for hypertension (*n* = 1144) and examined the associations of combined genetic and lifestyle risks with hypertension and home hypertension. Furthermore, to confirm the robustness of our results, we conducted an analysis of covariance and estimated the adjusted least-square means of SBP for 9 categories by genetic and lifestyle risk among participants without treatment for hypertension.

A two-sided *P* < 0.05 was considered statistically significant. Statistical analysis was performed using R version 4.1.2 (R Foundation for Statistical Computing, Vienna, Austria).

## Results

### Characteristics of participants

In the target data, the mean (SD) values for age, BMI, and Na/K ratio of participants were 57.8 (13.0) years, 22.7 (3.5) kg/m^2^, and 3.3 (0.7), respectively. The median (interquartile) number of home BP measurements was 13.0 [12.0, 14.0]. The mean (SD) values for SBP and DBP measured at the community support center were 128.6 (17.6) mmHg and 78.2 (10.6) mmHg, respectively. The mean (SD) values for home SBP and DBP were 127.2 (16.7) mmHg and 75.4 (10.1) mmHg, respectively. The proportion (%) of participants who were women, were non-obese, never consumed alcohol (never-drinkers), had regular physical activity, had low a Na/K ratio (less than 2.0), had office hypertension, and had home hypertension was 1097 (78.1%), 1082 (77.0%), 607 (43.3%), 636 (45.3%), 31 (2.2%), 570 (40.6%) and 566 (40.3%), respectively (Table, Supplemental Digital Content 4).

Similarly, the mean (SD) values for age, BMI, and Na/K ratio of the participants in test data were 58.1 (12.6) years, 22.7 (3.4) kg/m^2^, and 3.3 (0.7), respectively. The median (interquartile) number of home BP measurements was 13.0 [12.0, 14.0]. The mean (SD) values for SBP and DBP measured at the community support center were 128.6 (18.0) mmHg and 77.9 (10.9) mmHg, respectively. The mean (SD) values for home SBP and DBP were 127.1 (16.5) mmHg and 74.8 (9.9) mmHg, respectively. The proportion (%) of participants who were women, were non-obese, were never-drinkers, had regular physical activity, had low Na/K ratio (less than 2.0), had hypertension, and had home hypertension were 4317 (76.8%), 4376 (77.8%), 2470 (43.9%), 2360 (42.0%), 108 (1.9%), 2294 (40.8%) and 2322 (41.3%), respectively (Table 1). Notably, women tended to have healthier lifestyles than men.

**Table 1:** Characteristics of study participants according to combined genetic and lifestyle risks

Genetic risk	Low			Intermediate			High			All participants
Lifestyle	Ideal	Intermediate	Poor	Ideal	Intermediate	Poor	Ideal	Intermediate	Poor	
Number	352	807	715	295	787	792	234	740	900	5622
Age, years	59.8 (12.1)	57.9 (12.8)	57.8 (12.3)	61.5 (11.8)	59.1 (12.7)	56.4 (12.7)	60.5 (12.4)	58.3 (13.1)	56.4 (12.2)	58.1 (12.6)
Women, %	320 (90.9)	656 (81.3)	459 (64.2)	277 (93.9)	636 (80.8)	513 (64.8)	221 (94.4)	602 (81.4)	633 (70.3)	4317 (76.8)
BMI, kg/m^2^	21.3 (2.1)	21.8 (2.9)	24.3 (3.9)	21.2 (2.2)	22.0 (2.6)	24.0 (3.7)	21.2 (2.2)	21.9 (2.8)	23.8 (3.9)	22.7 (3.4)
SBP, mmHg	126.6 (17.7)	125.4 (17.8)	129.1 (17.3)	129.2 (17.1)	128.9 (18.9)	128.7 (18.2)	131.7 (19.0)	128.8 (18.0)	130.3 (17.7)	128.6 (18.0)
DBP, mmHg	74.8 (10.5)	75.3 (10.4)	79.3 (10.8)	76.3 (10.0)	77.5 (10.6)	79.2 (11.3)	77.5 (10.0)	77.4 (10.9)	80.2 (11.3)	77.9 (10.9)
Home SBP, mmHg	123.6 (15.4)	124.1 (16.1)	128.1 (16.8)	126.9 (16.3)	126.3 (16.3)	128.4 (16.5)	128.0 (16.4)	127.4 (16.2)	129.6 (16.9)	127.1 (16.5)
Home DBP, mmHg	71.1 (8.8)	75.3 (10.4)	76.4 (10.1)	73.2 (9.5)	73.9 (9.7)	76.8 (10.5)	73.3 (8.7)	74.5 (9.7)	77.1 (9.9)	74.8 (9.9)
Prevalence of hypertension	117 (33.2)	244 (30.2)	303 (42.4)	117 (39.7)	330 (41.9)	341 (43.1)	111 (47.4)	308 (41.6)	423 (47.0)	2294 (40.8)
Prevalence of home hypertension, %	104 (29.5)	253 (31.4)	321 (44.9)	125 (42.4)	308 (39.1)	357 (45.1)	96 (41.0)	315 (42.6)	443 (49.2)	2322 (41.3)
Treatment for hypertension, %	44 (12.5)	108 (13.4)	155 (21.7)	64 (21.7)	160 (20.3)	172 (21.7)	51 (21.8)	163 (22.0)	242 (26.9)	1169 (20.6)
Measurement time of home BP	13.0 [12.0, 14.0]	14.0 [12.0, 14.0]	13.0 [12.0, 14.0]	13.0 [12.0, 14.0]	14.0 [12.0, 14.0]	13.0 [12.0, 14.0]	13.5 [12.0, 14.0]	13.0 [12.0, 14.0]	13.0 [12.0, 14.0]	13.0 [12.0, 14.0]
Month (%)										
Summer	60 (17.0)	130 (16.1)	119 (16.6)	57 (19.3)	130 (16.5)	123 (15.5)	42 (17.9)	138 (18.6)	151 (16.8)	950 (16.9)
Winter	180 (51.1)	418 (5.18)	374 (52.3)	139 (47.1)	416 (52.9)	429 (54.2)	115 (49.1)	374 (50.5)	476 (52.9)	2921 (52.0)
Other	112 (31.8)	259 (32.1)	222 (31.0)	99 (21.7)	241 (30.6)	240 (30.3)	77 (32.9)	228 (30.8)	273 (30.3)	1751 (31.1)
Physical activity, MET-min/week	162.3 [56.8, 346.2]	76.5 [9.0, 207.3]	50.8 [8.7, 134.4]	177.0 [63.4, 362.0]	102.3 [16.8, 256.8]	45.0 [2.8, 133.3]	199.5 [53.4, 365.8]	89.1 [9.0, 244.4]	42.0 [2.8, 126.0]	71.8 [9.0, 198.0]
Sodium excretion, mEq/day	2.5 (1.1)	2.6 (1.1)	2.7 (1.2)	2.4 (1.0)	2.6 (1.1)	2.7 (1.2)	2.4 (1.2)	2.6 (1.2)	2.7 (1.1)	2.6 (1.1)
Potassium excretion, mEq/day	1.4 (0.9)	1.2 (0.7)	1.3 (0.7)	1.2 (0.7)	1.3 (0.7)	1.3 (0.7)	1.3 (0.9)	1.3 (0.7)	1.3 (0.7)	1.3 (0.8)
Sodium-to-potassium ratio	3.2 (0.8)	3.3 (0.7)	3.3 (0.7)	3.3 (0.8)	3.3 (0.7)	3.3 (0.7)	3.2 (0.8)	3.3 (0.7)	3.3 (0.7)	3.3 (0.7)
Drinking status, %										
Never-drinker	347 (98.6)	512 (63.4)	134 (18.7)	289 (98.0)	427 (54.3)	99 (12.5)	226 (96.6)	357 (48.2)	79 (8.8)	2,470 (43.9)
Ex-drinker	1 (0.3)	16 (2.0)	25 (3.5)	1 (0.3)	13 (1.7)	29 (3.7)	0 (0.0)	12 (1.6)	30 (3.3)	127 (2.3)
Current drinker	4 (1.1)	279 (34.6)	556 (77.8)	5 (1.7)	347 (44.1)	664 (83.8)	8 (3.4)	371 (50.1)	791 (87.9)	3,025 (53.8)
Healthy lifestyle factors										
Non-obesity	349 (99.1)	75 (9.3)	382 (53.4)	295 (100.0)	718 (91.2)	340 (42.9)	233 (99.6)	678 (91.6)	363 (40.3)	4,376 (77.8)
Never-drinker	347 (98.6)	512 (63.4)	134 (18.7)	289 (98.0)	427 (54.3)	99 (12.5)	226 (96.6)	357 (48.2)	79 (8.8)	2,470 (43.9)
Regular physical activity	333 (94.6)	362 (44.9)	73 (10.2)	288 (97.6)	421 (53.5)	104 (13.1)	225 (96.2)	429 (58.0)	125 (13.9)	2,360 (42.0)
Low-sodium-to-potassium ratio	33 (9.4)	8 (1.0)	2 (0.3)	16 (5.4)	8 (1.0)	1 (0.1)	23 (9.8)	16 (2.2)	1 (0.1)	108 (1.9)

*BMI* body mass index, *DBP* diastolic blood pressure, *MET* metabolic equivalent of task, *SBP* systolic blood pressure

Hypertension: defined as systolic/diastolic BP of 140/90 mmHg or higher measured at community-support center and/or self-reported treatment for hypertension

Home hypertension: defined as home systolic/diastolic BP of 135/85 mmHg or higher and/or self-reported treatment for hypertension

Month of measurements: Summer included June, July, August, and September. Winter included December, January, February, and March. Other months are categorized as others

Non-obesity: defined as BMI < 25.0 kg/m^2^ based on the Western Pacific Region of World Health Organization criteria for Japanese individuals

Never-drinker: defined as someone who consumes little or no alcohol or is constitutionally incapable of alcohol consumption using a self-reported questionnaire

Regular physical activity: defined as meeting the American Heart Association recommendations of at least 150 min of moderate activity per week or 75 min of vigorous activity per week

Low sodium-to-potassium ratio: defined as under 2.0

### Association of lifestyle with the prevalence of hypertension and home hypertension

A poor lifestyle was associated with an increased prevalence of hypertension and home hypertension. Compared with those of the ideal lifestyle group, the multivariable-adjusted ORs (95% CIs) for hypertension were 1.02 (0.86–1.21) and 1.47 (1.23–1.76) for the intermediate and poor lifestyle groups, respectively. Similarly, for home hypertension, the multivariable-adjusted ORs (95% CIs) were 1.11 (0.93–1.32) and 1.81 (1.51–2.17) for intermediate and poor lifestyle groups, respectively, compared with those of the ideal lifestyle group.

### Association of the PRS with the prevalence of hypertension and home hypertension

A higher PRS was associated with an increased OR for hypertension and home hypertension. In the multivariate model, for hypertension, the multivariate ORs (95% Cis) for low (reference), intermediate, and high were 1.00, 1.38 (1.19–1.59), and 1.69 (1.47–1.96), respectively, while for home hypertension, the respective values were 1.00, 1.33 (1.15–1.53), and 1.68 (1.45–1.94), respectively.

### Associations of genetic and lifestyle risk combination with the prevalence of hypertension and home hypertension

When genetic risk and lifestyle categories were combined, a monotonic relationship was found between increasing genetic risk and an increasingly unhealthy lifestyle (Tables 2 and 3 for hypertension and home hypertension, respectively). Participants with a low genetic risk and a poor lifestyle had significantly higher OR for the prevalence of hypertension and home hypertension than did those with a low genetic risk but an ideal lifestyle (OR 1.94 [95% CI, 1.34–2.80] for hypertension; OR 1.75 [95% CI, 1.20–2.54] for home hypertension). Participants with high genetic risk and an ideal lifestyle had significantly higher ORs for the prevalence of hypertension and home hypertension (OR 1.57 [95% CI, 1.17–2.10 for hypertension; OR 2.15 [95% CI, 1.60–2.90] for home hypertension). Participants with high genetic risk and a poor lifestyle had the highest ORs for the prevalence of hypertension and home hypertension (OR 2.29 [95% CI, 1.73–3.05] for hypertension; OR 3.08 [95% CI, 2.31–4.12] for home hypertension).

**Table 2:** Associations of genetic and lifestyle risk combination with the prevalence of hypertension

Genetic risk	Lifestyle category	Persons with HT/number of participants	%	OR, 95% CI	
Low	Ideal (≤1 poor factors)	117/352	(33.2)		Ref
	Intermediate (2 poor factors)	244/807	(30.2)	0.92	(0.67–1.23)
	Poor (≥3 poor factors)	303/715	(42.4)	1.57	(1.17–2.10)
Intermediate	Ideal (≤1 poor factors)	117/295	(39.7)	1.25	(0.88–1.77)
	Intermediate (2 poor factors)	330/787	(41.9)	1.53	(1.15–2.03)
	Poor (≥3 poor factors)	341/792	(43.1)	1.81	(1.36–2.42)
High	Ideal (≤1 poor factors)	111/234	(47.4)	1.94	(1.34–2.80)
	Intermediate (2 poor factors)	308/740	(41.5)	1.61	(1.21–2.15)
	Poor (≥3 poor factors)	423/900	(47.0)	2.29	(1.73–3.05)

*CI* confidence interval, *HT* hypertension, *OR* odds ratio

Adjusted for age, sex, and first six principal components

Hypertension: defined as systolic/diastolic BP of 140/90 mmHg or higher measured at community-support center and/or self-reported treatment for hypertension

**Table 3:** Associations of genetic and lifestyle risk combination with prevalence of home hypertension

Genetic risk	Lifestyle category	Persons with home HT/number of participants	%	OR, 95% CI	
Low	Ideal (≤1 poor factors)	104/352	(29.5)		Ref
	Intermediate (2 poor factors)	253/807	(31.4)	1.17	(0.87–1.57)
	Poor (≥3 poor factors)	321/715	(44.9)	2.15	(1.60–2.90)
Intermediate	Ideal (≤1 poor factors)	125/295	(42.4)	1.74	(1.23–2.46)
	Intermediate (2 poor factors)	308/787	(39.1)	1.60	(1.20–2.14)
	Poor (≥3 poor factors)	357/792	(45.1)	2.39	(1.78–3.22)
High	Ideal (≤1 poor factors)	96/234	(41.0)	1.75	(1.20–2.54)
	Intermediate (2 poor factors)	315/740	(42.6)	2.04	(1.52–2.74)
	Poor (≥3 poor factors)	443/900	(49.2)	3.08	(2.31–4.12)

*CI* confidence interval, *HT* hypertension, *OR* odds ratio

Adjusted for age, sex, first six principal components, and seasons of home BP measurements (summer, winter, and others)

Home hypertension: defined as home systolic/diastolic BP of 135/85 mmHg or higher and/or self-reported treatment for hypertension

### Comparison of the AUROC for models with and without PRS

Regarding hypertension, the AUROC value (95% CI) for the model that included lifestyle without a PRS was 0.743 (0.731–0.756), whereas that for the model with both lifestyle and a PRS was 0.748 (0.736–0.761) (*P* for difference <0.01). For home hypertension, the AUROC value (95% CI) for the model including lifestyle without a PRS was 0.749 (0.736–0.761), whereas that for the model with both lifestyle and a PRS was 0.753 (0.740–0.765) (*P* for difference <0.01) (Table 4).

**Table 4:** Area under the receiver operating characteristic curve for genetic and lifestyle risk to predict hypertension and home hypertension

	Area under the receiver operating characteristic curve (95% CI)	
	Hypertension	Home hypertension
Model 1^a^	0.739 (0.726–0.752)	0.741 (0.728–0.753)
Model 1 + lifestyle score	0.745 (0.732–0.758)	0.746 (0.733–0.758)
Model 1 + PRS	0.743 (0.731–0.756)	0.749 (0.736–0.761)
Model 1 + PRS + lifestyle score	0.748 (0.836–0.761)	0.753 (0.740–0.765)

*CI* confidence interval, *PRS* polygenic risk score

^a^Model 1 included age, sex, first six principal components for hypertension, and home BP only seasons of home BP measurements (summer, winter, and others) as predictors in the model

Hypertension: defined as systolic/diastolic BP of 140/90 mmHg or higher measured at community-support center and/or self-reported treatment for hypertension

Home hypertension: defined as home systolic/diastolic BP of 135/85 mmHg or higher and/or self-reported treatment for hypertension

The same pattern of associations was observed during sensitivity analysis, excluding participants with hypertension treatment (Tables, Supplemental Digital Content 5 and 6, for hypertension and home hypertension, respectively. Table, Supplemental Digital Content 7 shows the adjusted least-square means of SBP and home SBP, respectively).

## Discussion

In this general, community-based population of approximately 7000 Japanese individuals, the PRS, constructed based on clinical BP, was positively associated with the prevalence of hypertension and home hypertension, regardless of lifestyle risk. An unhealthy lifestyle was positively associated with the prevalence of hypertension and home hypertension, regardless of genetic risk. Thus, even in a population with low genetic risk, those with unhealthy lifestyles had significantly high ORs of hypertension and home hypertension. Nevertheless, participants with higher genetic risk also showed a higher prevalence of hypertension, even among participants with an ideal lifestyle.

The association between combined genetic and lifestyle risks and BP has been investigated. A cross-sectional study of the J-MICC showed that the PRS for BP was significantly associated with hypertension, independent of lifestyle factors, such as smoking, alcohol consumption, sedentary time, or obesity. Furthermore, these lifestyle factors were positively associated with the prevalence of hypertension, independent of genetic risk [19]. A follow-up with 4592 Chinese individuals showed that a high genetic risk and an unhealthy lifestyle were associated with elevated BP and risk of hypertension [18]. In the UK Biobank study, both genetic and lifestyle risks were associated with higher BP and risk of hypertension [16, 17]. Similarly, in our study, we found both genetic and lifestyle risks to be associated with a higher prevalence of hypertension, consistent with previous findings. AUROC values for the prevalence of hypertension and home hypertension were higher for models including a PRS than for those without. These findings suggest that the PRS of hypertension plays an additional role in home hypertension and hypertension beyond lifestyle risk factors. Therefore, considering a genetic risk may improve the ability to predict not only hypertension but also home hypertension. Since we have collected home BP follow-up data, we will perform a prospective cohort study to confirm whether the PRS constructed based on clinical BP can predict future home hypertension and improve the prediction ability.

We demonstrated that both genetic and lifestyle risks were associated with a higher prevalence of home hypertension. Home BP enables multiple measurements over a long period under relatively controlled conditions, eliminating observer and regression dilution biases [20, 21, 29, 30]. Home BP is a stronger index for the prediction of cerebrovascular and cardiovascular events than office BP [20–23]. Based on such evidence, several guidelines, including the United States Preventive Services Task Force, recommend obtaining BP measurements outside of the clinical setting for diagnostic confirmation for hypertension [4–7]. If a PRS constructed based on clinical BP can also predict home hypertension, it would hold significant implications, facilitating early identification and management of individuals at high risk for home hypertension. As we constructed a PRS based on a large GWAS of clinical BP in the Japanese population [28], which was also associated with home hypertension, our study adds to previous findings. Therefore, participants with high genetic risk should monitor their BP, irrespective of their lifestyle. The favorable association of lifestyle with home hypertension was independent of genetic risk, suggesting that even for cases of home hypertension, high genetic risk is mitigated by a favorable lifestyle.

To the best of our knowledge, this is the first study to show that a PRS constructed based on clinical BP is associated with home hypertension and hypertension as the TMMCommCohort Study is the largest genetic cohort dealing with home BP. Additionally, for the first time to our knowledge, we found that a healthy lifestyle score was associated with hypertension and home hypertension, regardless of the underlying genetic risk of BP. Given the decreasing cost of genetic analysis and the accumulation of further evidence on the influence of genetics and lifestyle on BP, this study is valuable as a piece of basic information in the realization of personalized prevention using own genetics.

Nevertheless, our study had some limitations. First, we could not confirm causal relationships as this was a cross-sectional study. A reverse causality of genetic factors is unlikely since genetic factors do not change owing to disease or lifestyle factors. However, lifestyle may be affected by a reverse causality. Furthermore, we assessed lifestyle as a composite score, thus, some participants classified as having ideal lifestyles may also have a few unhealthy lifestyles. Therefore, if participants have even a little bit of an unhealthy lifestyle, BP monitoring alone may be insufficient for the prevention of hypertension. We are currently collecting follow-up information on BP and will analyze similar relationships using home hypertension incident data. Therefore, we will follow up on the cohort to confirm causal relationships and whether a PRS constructed based on clinical BP can predict not only hypertension but also home hypertension. Second, lifestyle may be influenced by treatment for hypertension, which could have caused a reverse causality. However, our results remained substantially unchanged even after excluding participants receiving hypertension treatment. Third, our study population included only Japanese participants. Genetics, lifestyle, and the prevalence of hypertension vary by ethnicity [31, 32]. For example, Martin et al. [33] indicated that prediction accuracy for the PRS was consistently higher with GWAS summary statistics from ancestry-matched summary statistics. The J-MICC study showed that the PRS applying GWAS summary statistics from the Japanese population was positively associated with hypertension; however, a PRS constructed from the UK Biobank GWAS summary statistics was not associated with hypertension among the Japanese population [19]. Therefore, studies in other ethnic populations are required to confirm the generalizability of our findings. Fourth, additional variants associated with hypertension may be identified in future GWASs potentially improving genetic risk performance. Furthermore, rare variants explored by exome sequencing or whole genome sequencing may have effect modification in the association between lifestyle factors including salt intake and alcohol intake with BP. Further studies are warranted to elucidate the effect modification of the association between environment and BP due to genetics. The Tohoku Medical Megabank Project is ongoing to construct whole genome sequencing data for 100,000 participants and has already completed whole genome sequencing for more than 69,000 participants. We will examine whether the use of whole genome sequencing data improves the predictive ability of the PRS and elucidate the impact on genetics and lifestyle with BP. Fifth, we could not examine the association between genetic and lifestyle risk with the achievement of target blood pressure among participants with treatment for hypertension, since the number of those participants is limited. To elucidate it among hypertensive participants may provide valuable insight into the clinical setting.

### Perspective of Asia

Our findings support the notion that adhering to a healthy lifestyle such as avoiding obesity, salt reduction, increased potassium intake, exercise, and non-drink is important to prevent hypertension regardless of genetic risk. However, even among participants with a healthy lifestyle, PRS is positively associated with hypertension. Those findings are consistent with previous studies of China and Japan [18, 19]. However, since prospective cohort studies in Asia are scarce, further prospective confirmation studies on this topic are required.

## Conclusion

Our study provided a quantitative estimate of the relationship between combined genetic and lifestyle risks and the prevalence of hypertension and home hypertension in the general Japanese population. Our findings revealed that a PRS constructed based on clinical BP was associated with home hypertension and hypertension. A poor lifestyle was positively associated with hypertension and home hypertension, regardless of genetic risk. Therefore, our findings support the notion that adhering to a healthy lifestyle is important for preventing hypertension, regardless of genetic risk, and participants with high genetic risk should carefully monitor their BP, even if they adhere to a healthy lifestyle. To realize personalized medicine based on genetic and lifestyle information, further studies are necessary to clarify the clinical utility of PRS and the interaction between genetics and lifestyle.

## Supplementary information

Methods, Supplemental Digital Content 1

Supplementary Table1

Supplementary Figure 1

Supplementary Table 2

Supplementary Table 3

Supplementary Table 4

Supplementary Table 5

## Data Availability

The authors, Masato Takase and Atsushi Hozawa have full access to all data in the study and take responsibility for the integrity and accuracy of the data analysis.
