# Supplementary material for: Associations of combined genetic and lifestyle risks with hypertension and home hypertension
Source: Hypertens Res. 2024 Jun 24;47(8):2064–74. doi: 10.1038/s41440-024-01705-8 (PMC11298407; doi:10.1038/s41440-024-01705-8)
Supplement: Supplementary file 1 — Methods, Supplemental Digital Content 1 [file 41440_2024_1705_MOESM1_ESM.docx]

**Methods, Supplemental Digital Content 1**

***Healthy lifestyle factors***

Alcohol consumption status and physical activity were assessed using a self-reported questionnaire. Age was determined during a visit to the community support center. Based on the alcohol consumption status, participants were categorized as never-drinker (had consumed little or no alcohol or were constitutionally incapable of alcohol consumption), ex-drinker (had stopped drinking alcohol), or current drinker. Regular physical activity was defined as moderate activity of at least 150 min per week or vigorous activity of at least 75 min per week [1].

Height was measured to the nearest 0.1 cm using a stadiometer (AD6400; A&D Co., Ltd., Tokyo, Japan). Weight was measured in increments of 0.1 kg, and 1.0 kg was subtracted to account for the weight of the participant’s clothing using a body composition analyzer (InBody720; Biospace Co., Ltd., Seoul, Korea). The body mass index (BMI) was calculated as weight (kg) divided by height [meters squared (m^2^)]. Obesity was defined as BMI ≥25.0 kg/m^2^ based on the Western Pacific Region of World Health Organization criteria for Japanese [2].

Casual spot urine samples were collected from each participant. Estimates of 24-h urinary excretion of sodium and potassium from the spot urine samples were calculated using the Tanaka formula [3]. Subsequently, we calculated the sodium-to-potassium ratio (Na/K) as 24-h sodium excretion divided by 24-h potassium excretion. In previous studies, the urinary Na/K was strongly and positively associated with home hypertension, increasing the hypertension burden [4–6]. We set the cut-off values to the median (≥3.2).

As home blood pressure (BP) is affected by seasonal temperature changes [7, 8], we classified the timing of home BP measurement into the following three seasons: summer, winter, and others. For the classification of seasons, the average temperature of Sendai City from 2016 to 2018 was used as a representative of the Miyagi Prefecture. In descending order of monthly average temperature, we defined June, July, August, and September as summer, December, January, February, and March as winter, and the other months as other seasons.

Information on physical activity was collected using a self-reported questionnaire. Participants answered questions on the hours spent for each activity (sitting, standing, walking, and strenuous work) per average day in the last years [9]. We assigned 0, 30, 120, 240, 360, 480, 600, and 660 min to none, <1 h, 1–3 h, 3–5 h, 5–7 h, 7–9 h, 9–11 h, and ≥11 h, respectively. The average frequency (times/week) and duration (min/time) of normal walking, brisk walking, moderate-intensity exercise, and high-intensity exercise during leisure were obtained [9–11]. The frequency was classified into the following categories: less than once per month, 1–3 times per month, 1–2 times per week, 3–4 times per week, and almost every day. The duration was also classified into the following categories: <30 min, 30–59 min, 1–2 h, 2–3 h, 3–4 h, and ≥4 h. This study defined “walking,” “normal walking,” “brisk walking,” and “moderate-intensity exercise” as moderate physical activity and “strenuous work” and “high-intensity exercise” as vigorous physical activity [9–12]. The average time of moderate and vigorous physical activities in leisure time was determined by multiplying frequency and duration. Subsequently, we calculated the minutes of moderate-intensity activity per week by adding the duration of walking and the average time of moderate physical activity during leisure time. Similarly, we calculated the minutes of vigorous physical activity per week by adding the duration of strenuous work and the average time of vigorous physical activity during leisure time.

Participants answered whether they consumed alcohol at least once a month. We classified drinking status as “never-drinker” (participants who answered little or no drinking or could not drink constitutionally) and “drinker” (participants who answered stopped drinking or currently drinking”).

***Genotyping and quality control (QC)***

Study participants were genotyped using an Affymetrix Axiom Japonica Array (v2; Affymetrix, Santa Clara, CA, USA) separately in 21 batches [13, 14]. The direct genotype data were pre-phased using SHAPEIT2 [15], and the phased genotypes were subsequently imputed using IMPUTE 4 [16], with a cross-imputed haplotype reference panel of the 3.5KJPNv2 [17] and the 1000 Genomes phase 3 panel [18]. The cross-imputation of the two reference panels was executed using IMPUTE2 [19] with the -merge_ref_panels_output_ref option. For QC, we excluded plates with an average call rate <0.95 and removed samples with dish QC metric <0.82 or step 1 call rate <0.97 before batch genotyping. Next, we removed variants with a P-value of the Hardy–Weinberg Equilibrium test <1.00 × 10^-6^, a minor allele frequency (MAF) <0.01, or missing rate >0.01 from each batch. We merged the imputed genotype datasets for 21 batches using QCTOOL (v2.0.4) (https://www.well.ox.ac.uk~gavqctool) and obtained imputed and direct genotype datasets in Oxford BGEN and PLINK BED formats, respectively, from Tohoku Medical Megabank Community-based Cohort study participants. We applied QC to the imputed genotype dataset to remove variants with an MAF value <0.01 and IMPUTE2 info score <0.8.

**References**

1. Lloyd-Jones DM, Hong Y, Labarthe D, Mozaffarian D, Appel LJ, Van Horn L, et al. Defining and setting national goals for cardiovascular health promotion and disease reduction: the American Heart Association’s strategic Impact Goal through 2020 and beyond. Circulation 2010; 121: 586–613.

2. Iwahori T, Miura K, Obayashi K, Ohkubo T, Nakajima H, Shiga T, et al. Seasonal variation in home blood pressure: findings from nationwide web-based monitoring in Japan. BMJ Open 2018; 8: e017351.

3. Tanaka T, Okamura T, Miura K, Kadowaki T, Ueshima H, Nakagawa H, et al. A simple method to estimate populational 24-h urinary sodium and potassium excretion using a casual urine specimen. J Hum Hypertens 2002; 16: 97–103.

4. Kogure M, Hirata T, Nakaya N, Tsuchiya N, Nakamura T, Narita A, et al. Multiple measurements of the urinary sodium-to-potassium ratio strongly related home hypertension: TMM Cohort Study. Hypertens Res 2020; 43: 62–71.

5. Kogure M, Nakamura T, Tsuchiya N, Hirata T, Nochioka K, Narita A, et al. Consideration of the reference value and number of measurements of the urinary sodium-to-potassium ratio based on the prevalence of untreated home hypertension: TMM Cohort Study. Hypertens Res 2022; 45: 866–75.

6. Hirata T, Kogure M, Tsuchiya N, Miyagawa K, Narita A, Nochioka K, et al. Impacts of the urinary sodium-to-potassium ratio, sleep efficiency, and conventional risk factors on home hypertension in a general Japanese population. Hypertens Res 2021; 44: 858–65.

7. Bassett J, International Diabetes Institute, World Health Organization Regional Office for the Western Pacific, International Association for the Study of Obesity. The Asia-Pacific perspective: redefining obesity and its treatment. Australia: Health Communications Australia Pty Ltd; 2000.

8. Hozawa A, Kuriyama S, Shimazu T, Ohmori-Matsuda K, Tsuji I. Seasonal variation in home blood pressure measurements and relation to outside temperature in Japan. Clin Exp Hypertens 2011; 33: 153–8.

9. Fujii H, Yamamoto S, Takeda-Imai F, Inoue M, Tsugane S, Kadowaki T, et al. Validity and applicability of a simple questionnaire for the estimation of total and domain-specific physical activity. Diabetol Int 2011; 2: 47–54.

10. Kikuchi H, Inoue S, Lee IM, Odagiri Y, Sawada N, Inoue M, et al. Impact of moderate-intensity and vigorous-intensity physical activity on mortality. Med Sci Sports Exerc 2018; 50: 715–21.

11. Kikuchi H, Inoue S, Odagiri Y, Ihira H, Inoue M, Sawada N, et al. Intensity-specific validity and reliability of the Japan Public Health Center-based prospective study-physical activity questionnaire. Prev Med Rep 2020; 20: 101169.

12. MacIntosh BR, Murias JM, Keir DA, Weir JM. What is moderate to vigorous exercise intensity? Front Physiol 2021; 12: 682233.

13. Yamada M, Motoike IN, Kojima K, Fuse N, Hozawa A, Kuriyama S, et al. Genetic loci for lung function in Japanese adults with adjustment for exhaled nitric oxide levels as airway inflammation indicator. Commun Biol 2021; 4: 1288.

14. Fuse N, Sakurai M, Motoike IN, Kojima K, Takai-Igarashi T, Nakaya N, et al. Genome-wide association study of axial length in population-based cohorts in Japan: the Tohoku Medical Megabank Organization Eye Study. Ophthalmol Sci 2022; 2: 100113.

15. Delaneau O, Zagury JF, Marchini J. Improved whole-chromosome phasing for disease and population genetic studies. Nat Methods 2013; 10: 5–6.

16. Bycroft C, Freeman C, Petkova D, Band G, Elliott LT, Sharp K, et al. The UK biobank resource with deep phenotyping and genomic data. Nature 2018; 562: 203–9.

17. Tadaka S, Katsuoka F, Ueki M, Kojima K, Makino S, Saitō S, et al. 3.5KJPNv2: an allele frequency panel of 3552 Japanese individuals including the X chromosome. Hum Genome Var 2019; 6: 28.

18. 1000 Genomes Project Consortium, Auton A, Brooks LD, Durbin RM, Garrison EP, Kang HM et al. A global reference for human genetic variation. Nature 2015; 526: 68–74.

19. Howie BN, Donnelly P, Marchini J. A flexible and accurate genotype imputation method for the next generation of genome-wide association studies. PLOS Genet 2009; 5: e1000529.

**Table, Supplemental Digital Content 2. P-value threshold and related parameters for PRS construction using target data**

| **Traits** | **P-threshold** | **Number of SNPs** | **R^2^** |
| --- | --- | --- | --- |
| SBP | 5.0 × 10^-8^ | 48 | 0.003733 |
|  | **0.001** | **1,786** | **0.006080** |
|  | 0.01 | 9,299 | 0.002734 |
|  | 0.05 | 3,0964 | 0.003982 |
|  | 0.1 | 5,2305 | 0.004768 |
|  | 0.2 | 8,5526 | 0.005216 |
|  | 0.3 | 114,822 | 0.005651 |
|  | 0.4 | 140,133 | 0.004981 |
|  | 0.5 | 162,383 | 0.005594 |
| Home SBP | 5.0 × 10^-8^ | 48 | 0.004417 |
|  | **0.001** | **1,786** | **0.008605** |
|  | 0.01 | 9,299 | 0.002789 |
|  | 0.05 | 30,964 | 0.002412 |
|  | 0.1 | 52,305 | 0.003048 |
|  | 0.2 | 85,526 | 0.003218 |
|  | 0.3 | 114,822 | 0.004406 |
|  | 0.4 | 140,133 | 0.004400 |
|  | 0.5 | 162,383 | 0.004760 |

Bold values indicate the best fit and settings for our analysis.

PRS, polygenic risk score; SBP, systolic blood pressure; SNP, single nucleotide polymorphism

**Table, Supplemental Digital Content 4. Characteristics of study participants in target data**

| **Variables** | **All participants** |
| --- | --- |
| Number | 1,405 |
| Age, years | 57.8 (13.0) |
| Women, % | 1,097 (78.1) |
| BMI, kg/m^2^ | 22.7 (3.5) |
| SBP, mmHg | 128.6 (17.6) |
| DBP, mmHg | 78.2 (10.6) |
| Home SBP, mmHg | 127.2 (16.7) |
| Home DBP, mmHg | 75.4 (10.1) |
| Measurement time of home BP | 13.0 [12.0, 14.0] |
| Prevalence of hypertension | 570 (40.6) |
| Prevalence of home hypertension, % | 566 (40.3) |
| Treatment for hypertension, % | 277 (19.7) |
| Month (%) |  |
| Summer | 239 (17.0) |
| Winter | 723 (51.5) |
| Other | 443 (31.5) |
| Physical activity,  MET-min/week | 83.6 [14.8, 221.8] |
| Sodium excretion, mEq/day | 3.3 (0.7) |
| Potassium excretion, mEq/day | 1.3 (0.8) |
| Sodium-to-potassium ratio | 3.3 (0.7) |
| Drinking status, % |  |
| Never-drinker | 607 (43.2) |
| Ex-drinker | 36 (2.6) |
| Current drinker | 762 (54.2) |
| Healthy lifestyle factors |  |
| Non-obesity | 1,082 (77.0) |
| Never-drinker | 607 (43.2) |
| Regular physical activity | 636 (45.3) |
| Low-sodium-to-potassium ratio | 31 (2.2) |

BMI, body mass index; DBP, diastolic blood pressure; MET, metabolic equivalent of task; SBP, systolic blood pressure

**Table, Supplemental Digital Content 5.** **Associations of genetic and lifestyle risk combinations with the prevalence of hypertension among participants without treatment for hypertension**

| Genetic risk | Lifestyle category | Persons with HT/number of participants | % | OR, 95% CI | |
| --- | --- | --- | --- | --- | --- |
| Low | Ideal (≤1 poor factors) | 73/308 | (23.7) |  | Ref |
|  | Intermediate (2 poor factors) | 136/699 | (19.5) | 0.82 | (0.59-1.16) |
|  | Poor (≥3 poor factors) | 148/560 | (26.4) | 1.16 | (0.82-1.63) |
| Intermediate | Ideal (≤1 poor factors) | 53/231 | (22.9) | 0.97 | (0.64-1.47) |
|  | Intermediate (2 poor factors) | 170/627 | (27.1) | 1.28 | (0.92-1.78) |
|  | Poor (≥3 poor factors) | 169/620 | (27.3) | 1.32 | (0.95-1.86) |
| High | Ideal (≤1 poor factors) | 60/183 | (32.8) | 1.75 | (1.15-2.67) |
|  | Intermediate (2 poor factors) | 145/577 | (25.1) | 1.19 | (0.85-1.67) |
|  | Poor (≥3 poor factors) | 181/658 | (27.5) | 1.48 | (1.07-2.07) |

Hypertension was defined as systolic/diastolic BP of 140/90 mmHg or higher measured at the community support center.

Analysis using multivariate logistic regression model.

Adjusted for age, sex, and first six principal components.

BP, blood pressure; CI, confidence interval; HT, hypertension; OR, odds ratio

**able, Supplemental Digital Content 6. Associations of genetic and lifestyle risk combinations with the prevalence of home hypertension among participants without treatment for hypertension**

| Genetic risk | Lifestyle category | Persons with home HT/number of participants | % | OR, 95% CI | |
| --- | --- | --- | --- | --- | --- |
| Low | Ideal (≤1 poor factors) | 60/308 | (19.5) |  | Ref |
|  | Intermediate (2 poor factors) | 145/699 | (20.7) | 1.15 | (0.81-1.63) |
|  | Poor (≥3 poor factors) | 166/560 | (29.6) | 1.79 | (1.26-2.56) |
| Intermediate | Ideal (≤1 poor factors) | 61/231 | (26.4) | 1.53 | (1.00-2.33) |
|  | Intermediate (2 poor factors) | 148/627 | (23.6) | 1.34 | (0.95-1.91) |
|  | Poor (≥3 poor factors) | 185/620 | (29.8) | 1.94 | (1.37-2.77) |
| High | Ideal (≤1 poor factors) | 45/184 | (24.6) | 1.52 | (0.96-2.39) |
|  | Intermediate (2 poor factors) | 152/577 | (26.3) | 1.66 | (1.17-2.37) |
|  | Poor (≥3 poor factors) | 201/658 | (30.5) | 2.26 | (1.61-3.21) |

Home hypertension is defined as home systolic/diastolic BP of 135/85 mmHg or higher.

Analysis using multivariate logistic regression model.

Adjusted for age, sex, first six principal components, and seasons of home BP measurements (summer, winter, and others).

BP, blood pressure; CI, confidence interval; HT, hypertension; OR, odds ratio

**Table, Supplemental Digital Content 7. The adjusted least-square means of SBP for 9 categories by genetic and lifestyle risk among participants without treatment for hypertension.**

| Genetic risk | Lifestyle category | SBP | | Home SBP | |
| --- | --- | --- | --- | --- | --- |
|  |  | LS means | 95%CI | LS means | 95%CI |
| Low | Ideal (≤1 poor factors) | 122 | (121-124) | 126 | (124-128) |
|  | Intermediate (2 poor factors) | 123 | (122-124) | 125 | (124-127) |
|  | Poor (≥3 poor factors) | 126 | (125-127) | 128 | (127-130) |
| Intermediate | Ideal (≤1 poor factors) | 125 | (123-127) | 127 | (125-130) |
|  | Intermediate (2 poor factors) | 125 | (124-126) | 128 | (127-129) |
|  | Poor (≥3 poor factors) | 127 | (126-128) | 129 | (128-130) |
| High | Ideal (≤1 poor factors) | 127 | (124-129) | 131 | (128-133) |
|  | Intermediate (2 poor factors) | 126 | (125-127) | 129 | (127-130) |
|  | Poor (≥3 poor factors) | 128 | (127-129) | 130 | (129-132) |

Analysis using an analysis of covariance.

Adjusted for age, sex, first six principal components, and seasons of home BP measurements (summer, winter, and others) for home BP only.

BP, blood pressure; CI, confidence interval; HT, hypertension; LS means, least-square means
