# Supplementary material for: Associations of combined genetic and lifestyle risks with hypertension and home hypertension
Source: Hypertens Res. 2024 Jun 24;47(8):2064–74. doi: 10.1038/s41440-024-01705-8 (PMC11298407; doi:10.1038/s41440-024-01705-8)
Supplement: Supplementary file 2 — Supplementary Table1 [file 41440_2024_1705_MOESM2_ESM.docx]

**Supplementary Table1. P-value threshold and related parameters for PRS construction using target data**

| **Traits** | **P-threshold** | **Number of SNPs** | **R^2^** |
| --- | --- | --- | --- |
| SBP | 5.0 × 10^-8^ | 48 | 0.003733 |
|  | **0.001** | **1,786** | **0.006080** |
|  | 0.01 | 9,299 | 0.002734 |
|  | 0.05 | 3,0964 | 0.003982 |
|  | 0.1 | 5,2305 | 0.004768 |
|  | 0.2 | 8,5526 | 0.005216 |
|  | 0.3 | 114,822 | 0.005651 |
|  | 0.4 | 140,133 | 0.004981 |
|  | 0.5 | 162,383 | 0.005594 |
| Home SBP | 5.0 × 10^-8^ | 48 | 0.004417 |
|  | **0.001** | **1,786** | **0.008605** |
|  | 0.01 | 9,299 | 0.002789 |
|  | 0.05 | 30,964 | 0.002412 |
|  | 0.1 | 52,305 | 0.003048 |
|  | 0.2 | 85,526 | 0.003218 |
|  | 0.3 | 114,822 | 0.004406 |
|  | 0.4 | 140,133 | 0.004400 |
|  | 0.5 | 162,383 | 0.004760 |

Bold values indicate the best fit and settings for our analysis.

PRS, polygenic risk score; SBP, systolic blood pressure; SNP, single nucleotide polymorphism
