# Supplementary material for: Associations of combined genetic and lifestyle risks with hypertension and home hypertension
Source: Hypertens Res. 2024 Jun 24;47(8):2064–74. doi: 10.1038/s41440-024-01705-8 (PMC11298407; doi:10.1038/s41440-024-01705-8)
Supplement: Supplementary file 3 — Supplementary Figure 1 [file 41440_2024_1705_MOESM3_ESM.pdf]

Supplementary Figure 1. Flowchart of the statistical analysis in this study

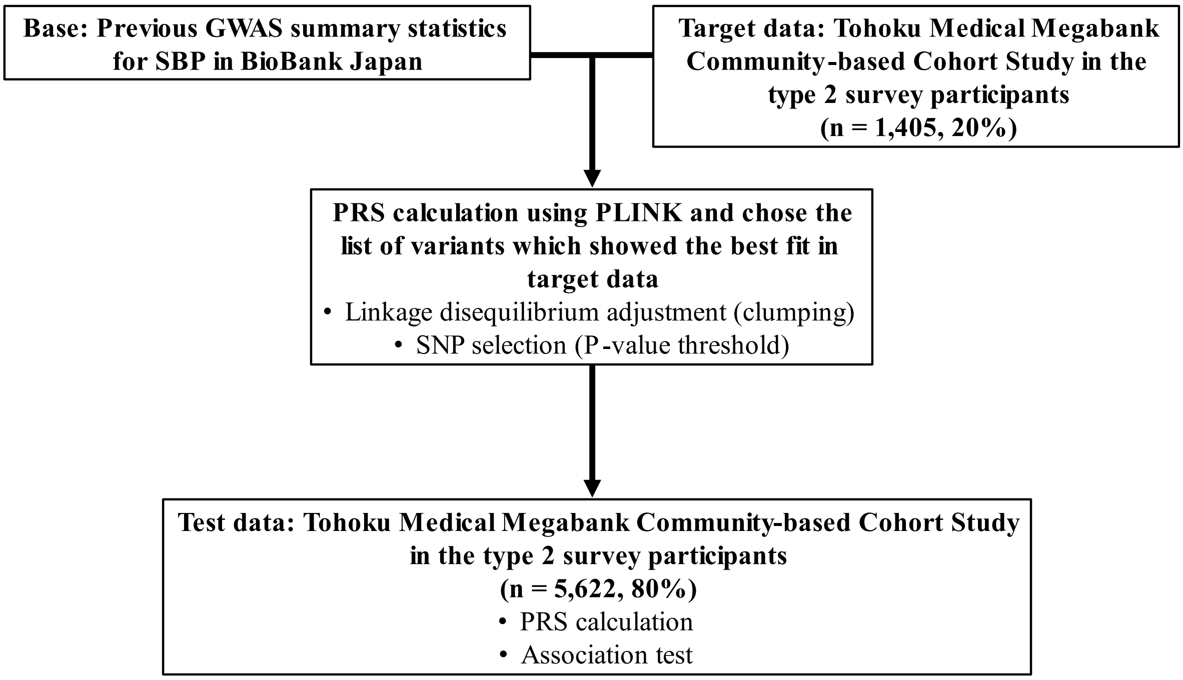

GWAS, genome-wide association study; PRS, polygenic risk score; SBP, systolic blood pressure; SNP, single nucleotide polymorphism
