# Supplementary material for: Associations of combined genetic and lifestyle risks with hypertension and home hypertension
Source: Hypertens Res. 2024 Jun 24;47(8):2064–74. doi: 10.1038/s41440-024-01705-8 (PMC11298407; doi:10.1038/s41440-024-01705-8)
Supplement: Supplementary file 4 — Supplementary Table 2 [file 41440_2024_1705_MOESM4_ESM.docx]

**Supplementary Table 2. Characteristics of study participants in target data**

| **Variables** | **All participants** |
| --- | --- |
| Number | 1,405 |
| Age, years | 57.8 (13.0) |
| Women, % | 1,097 (78.1) |
| BMI, kg/m^2^ | 22.7 (3.5) |
| SBP, mmHg | 128.6 (17.6) |
| DBP, mmHg | 78.2 (10.6) |
| Home SBP, mmHg | 127.2 (16.7) |
| Home DBP, mmHg | 75.4 (10.1) |
| Measurement time of home BP | 13.0 [12.0, 14.0] |
| Prevalence of hypertension | 570 (40.6) |
| Prevalence of home hypertension, % | 566 (40.3) |
| Treatment for hypertension, % | 277 (19.7) |
| Month (%) |  |
| Summer | 239 (17.0) |
| Winter | 723 (51.5) |
| Other | 443 (31.5) |
| Physical activity,  MET-min/week | 83.6 [14.8, 221.8] |
| Sodium excretion, mEq/day | 3.3 (0.7) |
| Potassium excretion, mEq/day | 1.3 (0.8) |
| Sodium-to-potassium ratio | 3.3 (0.7) |
| Drinking status, % |  |
| Never-drinker | 607 (43.2) |
| Ex-drinker | 36 (2.6) |
| Current drinker | 762 (54.2) |
| Healthy lifestyle factors |  |
| Non-obesity | 1,082 (77.0) |
| Never-drinker | 607 (43.2) |
| Regular physical activity | 636 (45.3) |
| Low-sodium-to-potassium ratio | 31 (2.2) |

BMI, body mass index; DBP, diastolic blood pressure; MET, metabolic equivalent of task; SBP, systolic blood pressure
