# Supplementary material for: Associations of combined genetic and lifestyle risks with hypertension and home hypertension
Source: Hypertens Res. 2024 Jun 24;47(8):2064–74. doi: 10.1038/s41440-024-01705-8 (PMC11298407; doi:10.1038/s41440-024-01705-8)
Supplement: Supplementary file 5 — Supplementary Table 3 [file 41440_2024_1705_MOESM5_ESM.docx]

**Supplementary Table 3.** **Associations of genetic and lifestyle risk combinations with the prevalence of hypertension among participants without treatment for hypertension**

| Genetic risk | Lifestyle category | Persons with HT/number of participants | % | OR, 95% CI | |
| --- | --- | --- | --- | --- | --- |
| Low | Ideal (≤1 poor factors) | 73/308 | (23.7) |  | Ref |
|  | Intermediate (2 poor factors) | 136/699 | (19.5) | 0.82 | (0.59-1.16) |
|  | Poor (≥3 poor factors) | 148/560 | (26.4) | 1.16 | (0.82-1.63) |
| Intermediate | Ideal (≤1 poor factors) | 53/231 | (22.9) | 0.97 | (0.64-1.47) |
|  | Intermediate (2 poor factors) | 170/627 | (27.1) | 1.28 | (0.92-1.78) |
|  | Poor (≥3 poor factors) | 169/620 | (27.3) | 1.32 | (0.95-1.86) |
| High | Ideal (≤1 poor factors) | 60/183 | (32.8) | 1.75 | (1.15-2.67) |
|  | Intermediate (2 poor factors) | 145/577 | (25.1) | 1.19 | (0.85-1.67) |
|  | Poor (≥3 poor factors) | 181/658 | (27.5) | 1.48 | (1.07-2.07) |

Hypertension was defined as systolic/diastolic BP of 140/90 mmHg or higher measured at the community support center.

Analysis using multivariate logistic regression model.

Adjusted for age, sex, and first six principal components.

BP, blood pressure; CI, confidence interval; HT, hypertension; OR, odds ratio
