# Supplementary material for: Associations of combined genetic and lifestyle risks with hypertension and home hypertension
Source: Hypertens Res. 2024 Jun 24;47(8):2064–74. doi: 10.1038/s41440-024-01705-8 (PMC11298407; doi:10.1038/s41440-024-01705-8)
Supplement: Supplementary file 6 — Supplementary Table 4 [file 41440_2024_1705_MOESM6_ESM.docx]

**Supplementary Table 4. Associations of genetic and lifestyle risk combinations with the prevalence of home hypertension among participants without treatment for hypertension**

| Genetic risk | Lifestyle category | Persons with home HT/number of participants | % | OR, 95% CI | |
| --- | --- | --- | --- | --- | --- |
| Low | Ideal (≤1 poor factors) | 60/308 | (19.5) |  | Ref |
|  | Intermediate (2 poor factors) | 145/699 | (20.7) | 1.15 | (0.81-1.63) |
|  | Poor (≥3 poor factors) | 166/560 | (29.6) | 1.79 | (1.26-2.56) |
| Intermediate | Ideal (≤1 poor factors) | 61/231 | (26.4) | 1.53 | (1.00-2.33) |
|  | Intermediate (2 poor factors) | 148/627 | (23.6) | 1.34 | (0.95-1.91) |
|  | Poor (≥3 poor factors) | 185/620 | (29.8) | 1.94 | (1.37-2.77) |
| High | Ideal (≤1 poor factors) | 45/184 | (24.6) | 1.52 | (0.96-2.39) |
|  | Intermediate (2 poor factors) | 152/577 | (26.3) | 1.66 | (1.17-2.37) |
|  | Poor (≥3 poor factors) | 201/658 | (30.5) | 2.26 | (1.61-3.21) |

Home hypertension is defined as home systolic/diastolic BP of 135/85 mmHg or higher.

Analysis using multivariate logistic regression model.

Adjusted for age, sex, first six principal components, and seasons of home BP measurements (summer, winter, and others).

BP, blood pressure; CI, confidence interval; HT, hypertension; OR, odds ratio
