# Supplementary material for: Associations of combined genetic and lifestyle risks with hypertension and home hypertension
Source: Hypertens Res. 2024 Jun 24;47(8):2064–74. doi: 10.1038/s41440-024-01705-8 (PMC11298407; doi:10.1038/s41440-024-01705-8)
Supplement: Supplementary file 7 — Supplementary Table 5 [file 41440_2024_1705_MOESM7_ESM.docx]

**Supplementary Table 5. The adjusted least-square means of SBP for 9 categories by genetic and lifestyle risk among participants without treatment for hypertension.**

| Genetic risk | Lifestyle category | SBP | | Home SBP | |
| --- | --- | --- | --- | --- | --- |
|  |  | LS means | 95%CI | LS means | 95%CI |
| Low | Ideal (≤1 poor factors) | 122 | (121-124) | 126 | (124-128) |
|  | Intermediate (2 poor factors) | 123 | (122-124) | 125 | (124-127) |
|  | Poor (≥3 poor factors) | 126 | (125-127) | 128 | (127-130) |
| Intermediate | Ideal (≤1 poor factors) | 125 | (123-127) | 127 | (125-130) |
|  | Intermediate (2 poor factors) | 125 | (124-126) | 128 | (127-129) |
|  | Poor (≥3 poor factors) | 127 | (126-128) | 129 | (128-130) |
| High | Ideal (≤1 poor factors) | 127 | (124-129) | 131 | (128-133) |
|  | Intermediate (2 poor factors) | 126 | (125-127) | 129 | (127-130) |
|  | Poor (≥3 poor factors) | 128 | (127-129) | 130 | (129-132) |

Analysis using an analysis of covariance.

Adjusted for age, sex, first six principal components, and seasons of home BP measurements (summer, winter, and others) for home BP only.

BP, blood pressure; CI, confidence interval; HT, hypertension; LS means, least-square means
